# MMP12-dependent myofibroblast formation contributes to nucleus pulposus fibrosis

**DOI:** 10.1172/jci.insight.180809

**Published:** 2025-03-04

**Authors:** Yi Sun, Wai-Kit Tam, Manyu Zhu, Qiuji Lu, Mengqi Yu, Yuching Hsu, Peng Chen, Peng Zhang, Minmin Lyu, Yongcan Huang, Zhaomin Zheng, Xintao Zhang, Victor Y. Leung

**Affiliations:** 1 Department of Sports Medicine, Peking University Shenzhen Hospital, Shenzhen, China.; 2 Department of Orthopaedics & Traumatology, The University of Hong Kong, Hong Kong SAR, China.; 3 The University of Hong Kong-Shenzhen Hospital, Shenzhen, China.; 4 Department of Spine Surgery, Peking University Shenzhen Hospital, Shenzhen, China.; 5 Department of Spine Surgery, the First Affiliated Hospital, Sun Yat-sen University, Guangzhou, China.

**Keywords:** Bone biology, Cell biology, Cartilage, Fibrosis

## Abstract

Intervertebral disc degeneration (IDD) is associated with low back pain, a leading cause of disability worldwide. Fibrosis of nucleus pulposus (NP) is a principal component of IDD, featuring an accumulation of myofibroblast-like cells. Previous study indicates that matrix metalloproteinase 12 (MMP12) expression is upregulated in IDD, but its role remains largely unexplored. We here showed that TGF-β1 could promote myofibroblast-like differentiation of human NP cells along with an induction of MMP12 expression. Intriguingly, MMP12 knockdown not only ameliorated the myofibroblastic phenotype but also increased chondrogenic marker expression. Transcriptome analysis revealed that the MMP12-mediated acquisition of myofibroblast phenotype was coupled to processes related to fibroblast activation and osteogenesis and to pathways mediated by MAPK and Wnt signaling. Injury induced mouse IDD showed NP fibrosis with marked increase of collagen deposition and αSMA-expressing cells. In contrast, MMP12-KO mice exhibited largely reduced collagen I and III but increased collagen II and aggrecan deposition, indicating an inhibition of NP fibrosis along with an enhanced cartilaginous matrix remodeling. Consistently, an increase of SOX9^+^ and CNMD^+^ but decrease of αSMA^+^ NP cells was found in the KO. Altogether, our findings suggest a pivotal role of MMP12 in myofibroblast generation, thereby regulating NP fibrosis in IDD.

## Abstract 2

<a>**MMP12 is important to myofibroblast formation during intervertebral disc fibrosis and degeneration**</a>

## Introduction

Low back pain is a major cause of disability, imposing tremendous socioeconomic burden worldwide. It is associated with intervertebral disc degeneration (IDD), which attributes to dysfunction of the central gelatinous nucleus pulposus (NP). NP matrix degradation and remodeling has been regarded a primary feature of IDD (1). Accumulative evidence from studies of clinical samples and animal models indicates NP fibrosis and calcification in IDD with excessive deposition of fibronectin and collagen I and III (1–4). Together with reduced swelling pressure due to loss of proteoglycans, these changes compromise the capacity of the disc in withstanding loads, ultimately leading to spinal segment instability and pain (5).

Abnormal matrix deposition in tissue fibrosis is attributed to dysregulated myofibroblastic activity mediated by fibrogenic cytokines (6). TGF-β is regarded as the primary inducer of fibrosis via driving myofibroblast differentiation of mesenchymal cells, fibroblasts, pericytes, or macrophages by activating the Alk5/Smad3 pathway (7–10). Studies support that disc fibrosis and degeneration was mediated by TGF-β1 pathway, and suppression of TGF-β pathway by Alk5 inhibitor or halofuginone could attenuate IDD (11, 12). TGF-β alters marker expression in NP cells (NPC) (13). The presence of myofibroblastic cells, which are positive for α-smooth muscle actin (αSMA), together with an increased vimentin and decreased cytokeratin expression was reported in degenerative NP (dNP) (4). A lineage tracing study in a mouse injury-induced IDD model further suggested that fibroblastic cells could be derived from resident NPC (14). These results imply that NP fibrosis might be caused by a response of NPC to myofibroblastic differentiation under TGF-β signaling.

Matrix metalloproteinase 12 (MMP12) can regulate myofibroblast induction and be induced by TGF-β1 (15–17), contributing to pulmonary fibrosis through Bid-activated pathway (16) and profibrotic genes *EGR1* and *CYR61* (17). In IDD, higher expression of MMP12 in annulus fibrosus (AF) cells was found associated with increased disc deformity (18). Increased abundance of MMP12^+^ NPC coexpressing αSMA was also reported in human and sand rat IDD (4, 19). However, the regulatory role of MMP12 in NPC and IDD is yet to be elucidated. In this study, we investigated if MMP12 may regulate myofibroblast differentiation in human NPC and mediate the NP remodeling process and fibrosis in an injury-induced IDD model. Our results suggest a potentially new path to control NP fibrosis via targeting MMP12 expression.

## Results

**Figure 1:**
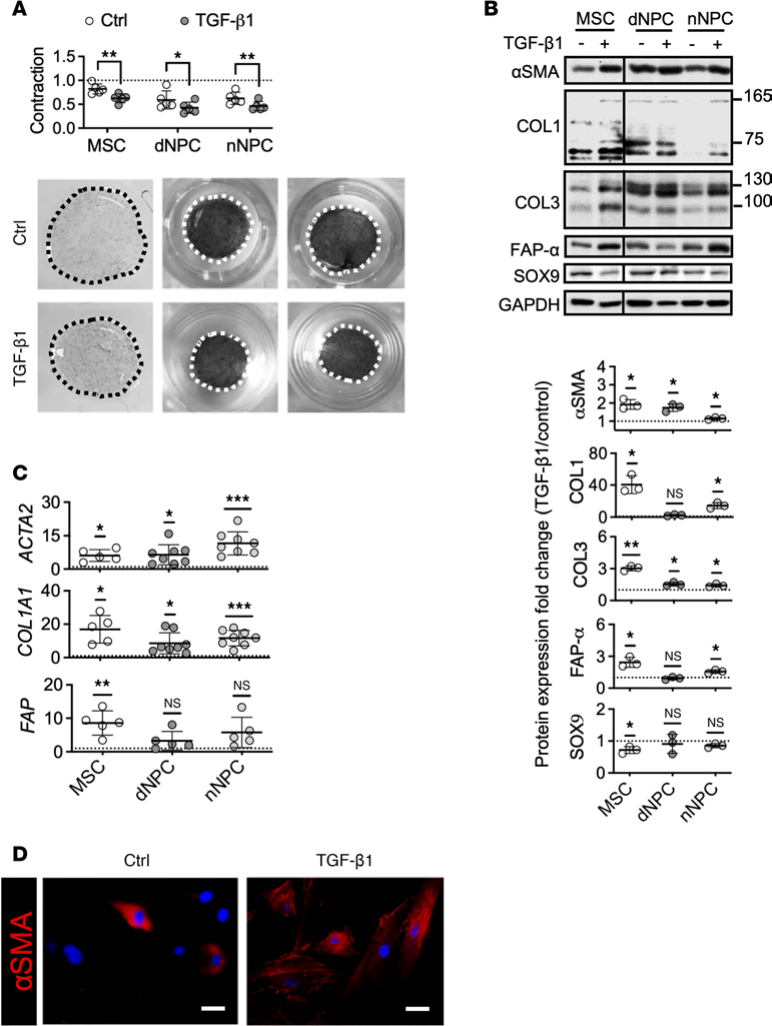
TGF-β1 induced myofibroblast-like differentiation of human NPC. (**A**) Collagen gel contraction of human MSC, nNPC, and dNPC and quantification as fold change of diameters of contracted gels to the culture wells (*n* = 5 biological replicates/group). (**B**) Immunoblotting for protein expression of αSMA, FAP-α, SOX9, and collagen I (COL1) and III (COL3) as well as semiquantitative analysis, expressed as fold changes of control (*n* = 3). (**C**) Reverse transcription PCR for myofibroblastic markers *ACTA2* (encoding αSMA), *FAP* (encoding FAP-α) and *COL1A1* (encoding collagen I α chain) (*n* = 5–8 per group). (**D**) Immunofluorescence of αSMA (red) in dNPC. Scale bar: 100 μm. All the experiments were repeated for 3 times. Data are expressed as the mean ± SD. *P < 0.05, **P < 0.01, ***P < 0.001, 1-sample *t* test for PCR and protein expression analysis; unpaired *t* test for gel contraction analysis.

We tested if TGF-β can induce fibroblast phenotype in human NPC. NPC from human degenerative (dNPC) and non-degenerative scoliotic (nNPC) disc tissues (less than passage 5, *n* = 5) were preconditioned in alginate for redifferentiation prior to TGF-β1 treatment (20). Collagen gel contraction assay in monolayer culture indicated that TGF-β1 could activate a contractile capacity, demonstrated by a pronounced size reduction in both dNPC (21%) and nNPC (17%) constructs (Figure 1A), a level comparable with that found in human bone marrow–derived mesenchymal stromal cells (BM-MSCs) (24%). Expression of αSMA (*ACTA2*), which marks myofibroblast maturation with cellular contractility (21), was substantially increased at both protein (Figure 1B) and mRNA (Figure 1C) levels in TGF-β1–treated MSCs and NPC, together with an upregulation of collagen I (*COL1A1*) and collagen III (*COL3A1*) expression. An increase in cytosolic αSMA positivity was noticed in TGF-β–treated dNPC (Figure 1D). TGF-β1 tended to increase FAP-α, a conventional biomarker for fibroblasts and miofibroblasts, although no statistical significance was found (Figure 1C). These findings implicate TGF-β can induce myofibroblast differentiation in human NPC. Notably, Sox9 expression levels in NPC was not altered by TGF-β1, possibly due to an attenuated prochondrogenic effect under the differentiated NPC state or the culture system, or related to the availability of additional cofactors (22).

**Figure 2:**
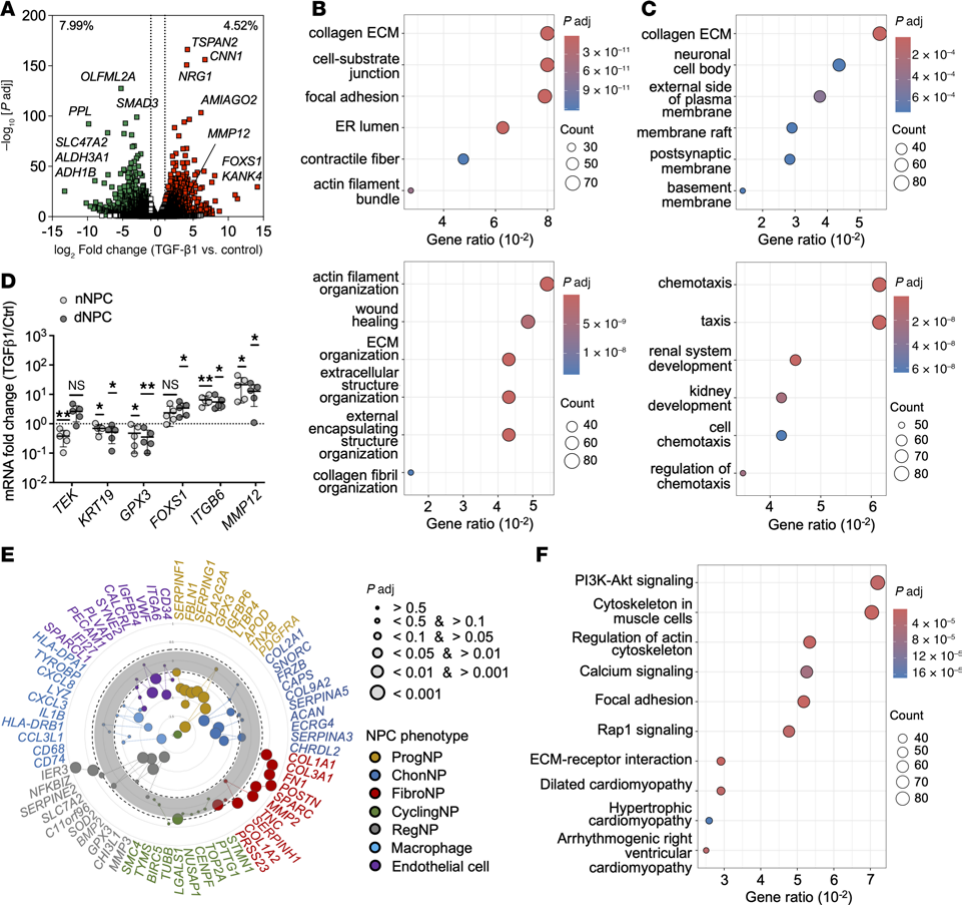
Transcriptomic analysis of TGF-β1–treated human dNPC. (**A**) Volcano plot of differentially expressed genes. (**B** and **C**) Gene Ontology of cellular components (upper) and biological processes (lower) for upregulated (**B**) and downregulated (**C**) DEGs. (**D**) qPCR for selected genes from transcriptome and represented as fold changes from nontreated NPC (*n* = 5 biologic replicates/group). Data are expressed as the mean ± SD. *P < 0.05, **P < 0.01 determined by 1-sample *t* test. (**E**) Scatter plot of scaled expression for previously defined biomarkers of various NPC subtypes. Genes names are provided in Supplemental Table 1. (**F**) KEGG pathway analysis.

We next performed RNA-Seq to interrogate how TGF-β1 modulated NPC phenotype (Figure 2). We found that 4.52% genes were upregulated after TGF-β1 treatment, which covered components related to cell-matrix interaction, contractile fiber, and collagen matrix, and enriched in processes linked to matrix organization, wound healing, and collagen fibril organization (Figure 2, A and B). In contrast, 7.99% genes were downregulated in which most of them related to chemotaxis, taxis, and cell adhesion (Figure 2C). Among the upregulated genes, *TSPAN2*, *FOXS1*, and *NOX4* have previously been linked to fibroblast activity. *TSPAN2* is enriched during cardiac fibroblast activation (23). *FOXS1* is a major regulator of fibroblast-to-myofibroblast differentiation in wound healing (24), and its expression was found substantially induced in TGF-β1–treated dNPC (Figure 2D). *NOX4* can mimic *GPX3* deficiency to activate myofibroblasts in kidney fibrosis (25). Notably, the expression of *GPX3*, which marks the regulatory NPC (26–28), was strongly reduced under the presence of TGF-β1. The expression of disc progenitor marker *TEK* (encoding Tie2) (29) was not altered in dNPC but was extensively reduced in TGF-β1–treated nNPC. Integrin subunit β6 (*ITGB6*), a marker of arteriovenous fistula myofibroblast (30), was substantially increased after treatment. Notably, TGF-β1 could strongly upregulate the expression of *MMP12* in the NPC. Inspecting the markers of various NPC subpopulations previously identified in single-cell study of human intervertebral disc (IVD) (26–28) indicated a remarkably phenotypic shift to the fibroblastic (FibroNP) and proliferating NPC (CyclingNP) states, whereas the progenitor NPC (ProgNP) and macrophage-like phenotype were reduced (Figure 2E and Supplemental Table 1; supplemental material available online with this article; https://doi.org/10.1172/jci.insight.180809DS1). As in myofibroblast differentiation of interstitial cells (8) and pericytes (7), an upregulation of PI3K/Akt pathway was found in the TGF-β1–treated dNPC (Figure 2F). Pathways related to focal adhesion and actin cytoskeleton were also highly enriched after treatment.

**Figure 3:**
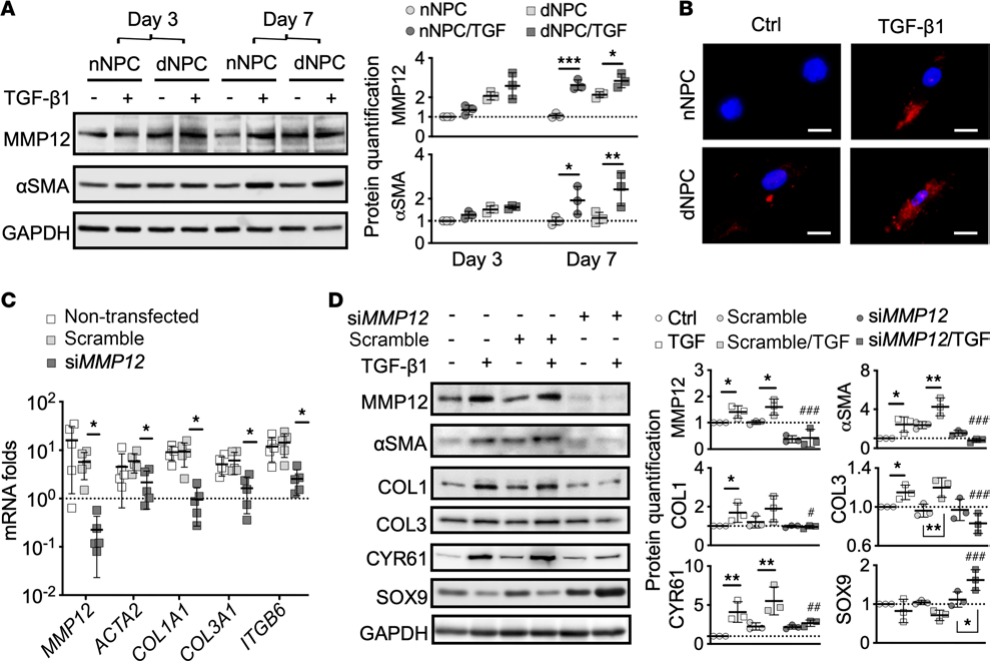
MMP12-dependent myofibroblast differentiation of human NPC. (**A**) Immunoblotting and densitometric quantification of MMP12 and αSMA expression in TGF-β1–induced myofibroblast differentiation of nNPC and human dNPC (*n* = 3). GAPDH is used as a loading control. (**B**) Immunofluorescence of MMP12. Scale bar: 100 μm. (**C** and **D**) RT-PCR and immunoblotting for MMP12, myofibroblastic markers αSMA (*ACTA2*), collagen I (*COL1A1*) and III (*COL3A1*), integrin subunit β6 (*ITGB6*), fibrotic regulator cysteine rich protein angiogenic inducer 61 (*CYR61*), and chondrogenic factor SOX9 in dNPC treated with TGF-β1 and transfected with *MMP12* siRNA (*siMMP12*). Semiquantitative analysis showing fold changes from nontreated NPC (*n* = 5 biological replicates/group). *P < 0.05, **P < 0.01 determined by Brown-Forsythe and Welch ANOVA with Dunnett’s T3 test for RT-PCR and repeated measures 2-way ANOVA with Bonferroni’s multiple-comparison test for protein quantification; ^#^*P* < 0.05, ^##^*P* < 0.05, ^###^*P* < 0.001 calculated by comparison between the scramble/TGF-β1 and the siMMP12/TGF-β1 groups.

We further validated the production of MMP12 and αSMA under TGF signaling. Consistent with the upregulation at the transcription level, TGF-β1 could concurrently promote MMP12 and αSMA protein expression in a time-dependent manner in the NPC (Figure 3A). Nuclear localization of MMP12 in addition to cytosolic expression was detected in dNPC but barely detectable in nNPC (Figure 3B). To examine whether MMP12 is essential to TGF-β1–mediated myofibroblast differentiation of NPC, we knocked down *MMP12* in dNPC by small interfering RNA (si*MMP12*; Figure 3, C and D). The si*MMP12* could largely reduce the TGF-β1–induced upregulation of αSMA (*ACTA2*) and collagen I (*COL1A1*) and III (*COL3A1*) at both mRNA (Figure 3C) and protein levels (Figure 3D). CYR61, a high level of which can indicate myofibroblastic activation (17), was downregulated by si*MMP12* in the NPC (Figure 3D). We also observed an inhibition of SOX9 expression in the TGF-β1–treated dNPC. Interestingly, the expression of SOX9 at the protein level could be reversed by si*MMP12* and increased approximately 1.45 fold. This suggests that a removal of MMP12 may recover the chondrogenic phenotype in NPC under the TGF-β1–mediated myofibroblast differentiation.

**Figure 4:**
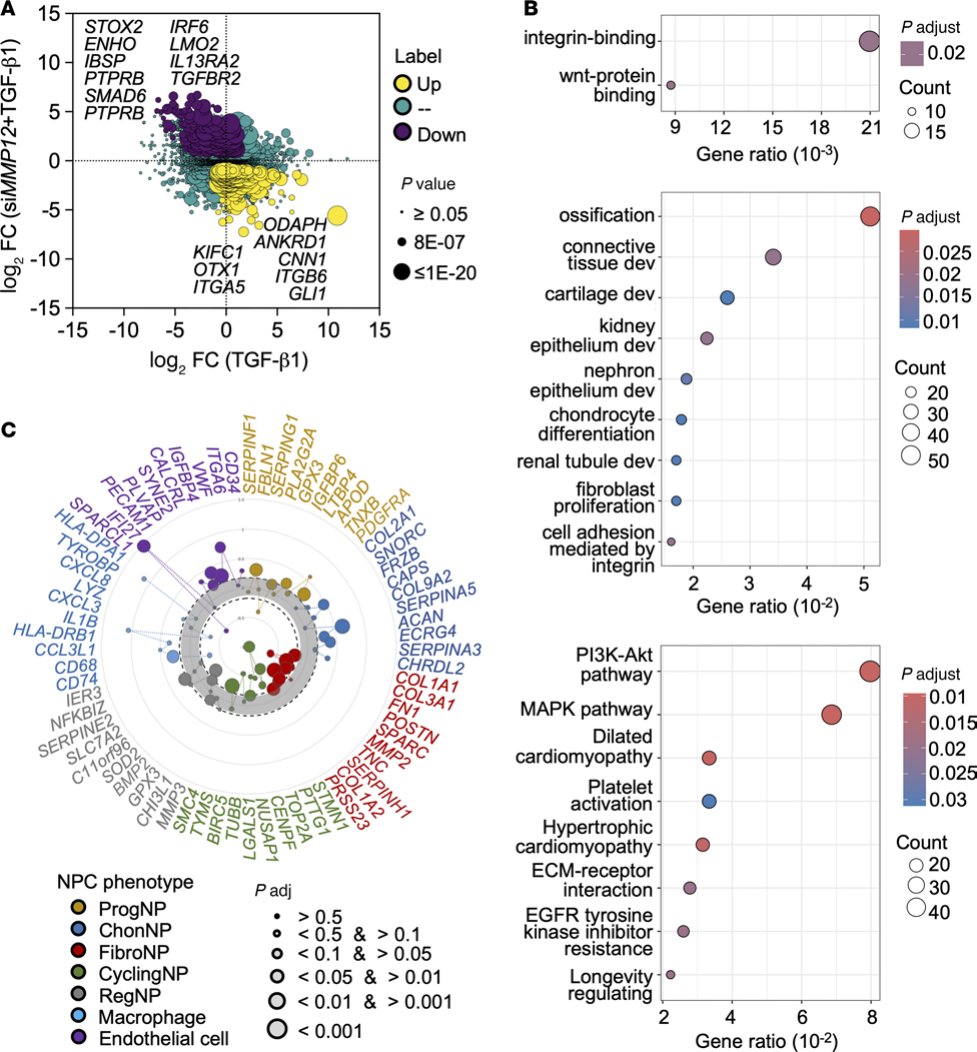
Pathway regulation by MMP12 in human dNPC. (**A**) Gene expression plot of TGF-β1–treated dNPC (*n* = 3 biologic replicates) with or without MMP12 knockdown. The *x* axis shows fold changes in cells with scramble transfection with TGF-β1 treatment; the *y* axis shows fold changes in TGF-β1–treated cells with *siMMP12* transfection. Differentially expressed genes (DEGs) with over 2-fold changes of expression at *P* ≤ 0.05 are colored in yellow (upregulated) or purple (downregulated). (**B**) Gene Ontology and KEGG pathway analysis of the DEGs: molecular function (upper); biological processes (middle); KEGG (lower). (**C**) Scatter plot for scaled expression of the previously defined biomarkers of various NPC subtypes. Genes between the dot lines (fold changes < 2) are considered nonsignificant. Genes names are provided in Supplemental Table 1.

To investigate the potential pathways regulated by MMP12, we examined the gene expression profile in the MMP12 knockdown NPC. Transcriptome study showed that MMP12 knockdown could negate the expression of a panel of genes upregulated by TGF-β1 in dNPC (Supplemental Table 1), including genes implicated in fibroblast activation such as *ITGB6* (30), *ANKRD1* (31), and *GLI1* (32) and genes related to osteogenesis such as *ODAPH* (33) (Figure 4A). *IBSP*, which could regulate osteoblast differentiation via PI3K/Akt (34), was markedly upregulated by MMP12 knockdown. Restoration of *ENHO* (32) and *IL13RA2* (35) expression in the MMP12-knockdown NPC suggested that the inhibition of fibroblastic differentiation may involve a deactivation of GLI1-dependent profibrogenic program. A number of TGF-β signaling components (e.g., *STOX2*, *TGFBR2*, and *SMAD6*) were found enriched in the MMP12 knockdown, suggesting a selective modulatory effect of MMP12 on TGF-β signaling. The most enriched genes in the MMP12 knockdown were related to ossification and fibroblast proliferation (Figure 4B). Genes related to Wnt protein binding were also enriched. Pathway analysis suggested a modulation of the PI3K/Akt and MAPK (Figure 4B). By cross-referencing with previously defined NPC subtype markers, MMP12-knockdown cells displayed a largely diminished FibroNP/CyclingNP phenotype (Figure 4C) and concomitantly enriched the ChonNP and endothelial phenotypes (Figure 4C). Altogether, these data imply that MMP12 is required for TGF-β–mediated myofibroblastic differentiation.

**Figure 5:**
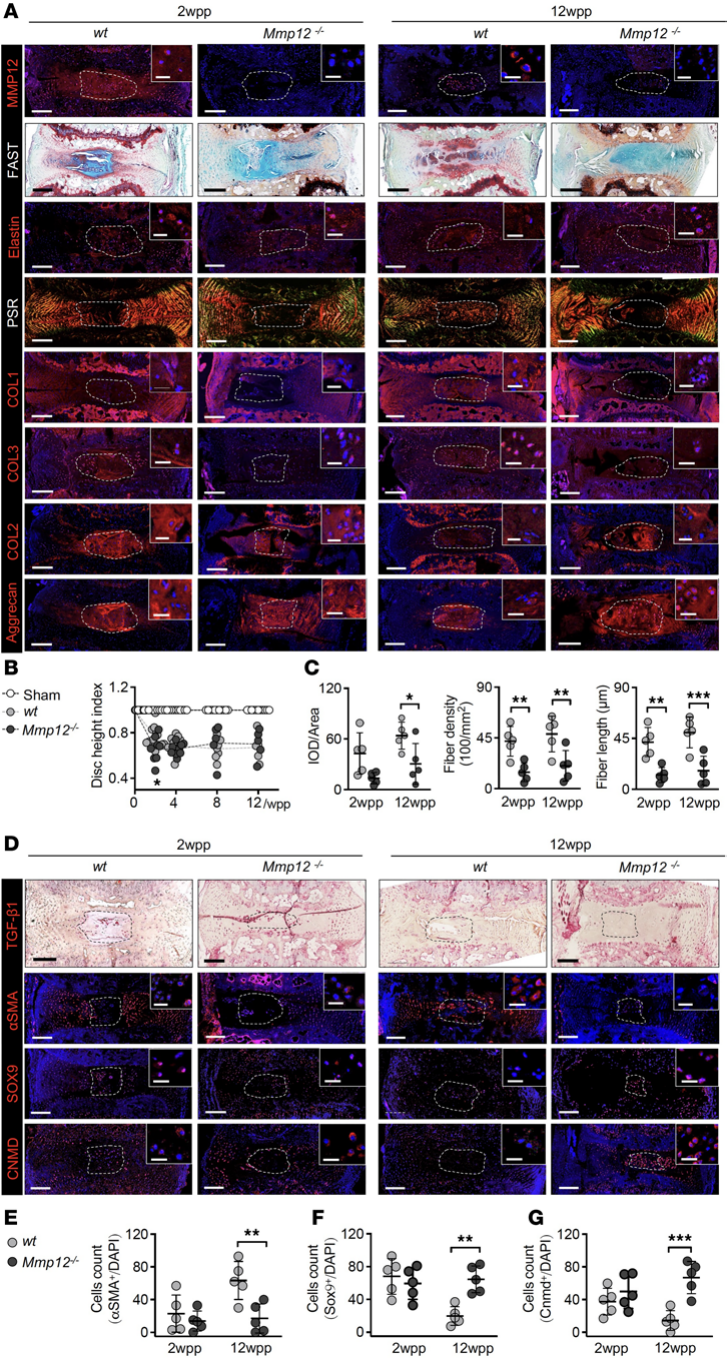
Inhibition of NP fibrosis and myofibroblast formation in *Mmp12^–/–^* mice. (**A**) Time course assessment of NP integrity and remodeling in punctured tail discs of WT and *Mmp12*-KO mice (*Mmp12^–/–^*) (*n* = 5 mice/group at each time point) by FAST staining, polarized microscopy of Picrosirius red staining (PSR), and immunofluorescence for collagen I (COL1), II (COL2), and III (COL3) as well as aggrecan proteoglycan. Expression of MMP12 and its substrate elastin were also examined. Cell nuclei are stained with DAPI. Bright red-yellow and green color in the PSR staining reflect collagen I and III containing fibers, respectively (39). NP regions are shown by dotted white line. Scale bar: 200 μm and 25 μm (insert). wpp, weeks postpuncture. (**B**) Disc height index calculated and represented as fold change from unoperated level. (**C**) Quantification of collagen fibers within the NP by CT-FIRE analysis based on the PSR staining signals: integrated optical density (IOD) of collagen I (red and yellow) and collagen III (green); collagen fiber density (× 100/mm^2^) and average fiber length (μm) (39). ROI, region of interest. (**D**) Detection of αSMA, SOX9, and CNMD expressing cells by immunofluorescence (*n* = 5 mice/group). NP regions are shown by dotted white line. Scale bar: 200 μm and 25 μm (insert). (**E**–**G**) Quantitative analysis of the αSMA^–^ (**E**), SOX9^–^ (**F**), and CNMD-expressing NPC (**G**) by counting of the positive cells and averaged to DAPI counts (indicative of total cell number) in the NP area (*n* = 5). *P < 0.01, **P < 0.01, ***P < 0.001 determined by 2-way ANOVA with Bonferroni post hoc test. Data are presented as the mean ± SD.

We next questioned if generation of myofibroblastic NPC in vivo requires MMP12. Annulus puncture was previously shown to induce mouse IDD with NP fibrosis and a fibroblastic transformation of resident NPC (4, 14). We found that MMP12 expression was robustly upregulated in the extracellular matrix at 2 weeks postpuncture (2wpp) and became intracellularly localized at 12wpp (MMP12; Figure 5A). Elastin, as a key substrate of MMP12 (36), was predominantly expressed in the cartilaginous endplate and inner AF (Supplemental Figure 1), and its expression was increased in the NP after puncture (Elastin, Figure 5A). While the *Mmp12*-deficient mice lost MMP12 expression in the NP and AF (*Mmp12^–/–^*; Figure 5A), they showed a comparable elastin expression and distribution in the matrix as opposed to the WT mice (Figure 5A). The WT mice showed a gradual substitution of Alcian blue^+^ matrix for Safranin O^+^ matrix in the NP in FAST staining after puncture (FAST; Figure 5A), indicating degenerative matrix remodeling (14, 37). Fissures were observed in the NP, annulus, and endplate, and NP-endplate boundaries became indiscernible at 12wpp. In contrast, the NP in the mutants presented a more intense Alcian blue^+^ matrix, showing a higher integrity score and a lower fibrosis score compared with the WT (Figure 5A and Supplemental Figure 2). Examination of IVD histological scores found no significant differences between the WT and mutants (Supplemental Figure 2). The disc heights in both WT and mutants were comparable despite an accelerated disc height loss at 2wpp (Figure 5B).

Picrosirius red staining under polarized light microscopy indicated disrupted collagen orientation with accumulation of thick collagen fibers at an early degeneration stage in the WT NP, whereas thinner fibers and delayed fiber maturation (38) was found in the mutants (Figure 5A). Quantitative analysis showed that the abundance, density and average length of collagen bundles in the NP were significantly lower in the mutant mice (Figure 5C and Supplemental Figure 3). Specifically, collagen I and III deposition were found progressively increased in the NP and AF after puncture in the WT but was largely suppressed in the mutants (COL1; Figure 5A). Collagen II and aggrecan proteoglycans are 2 key matrix components in mature human NP. Both matrix proteins were found expressed in the early phase of NP remodeling, yet they gradually decreased at later stage (COL2 and aggrecan; Figure 5A). By contrast, they were robustly expressed in the mutant NP and inner AF and persisted up to 12wpp (Figure 5A), implying that removal of MMP12 can promote chondrogenic differentiation while reducing fibrosis. Supporting this notion, emergence of αSMA^+^ cells in the WT NP was observed after puncture, constituting up to 62.5% NPC at 12wpp, and this was largely reduced in the mutant NP (19.4%) (Figure 5, D and E). The majority of WT NPC at early degeneration were positive to SOX9 or CNMD (Figure 5D), which were decreased to less than 20% at late degeneration (Figure 5, F and G). However, the mutants maintained a much higher level (over 60%) of SOX9^+^ and CNMD^+^ NPC at 12wpp (Figure 5, F and G). TGF-β1 appeared invariably expressed in both WT and mutant NP or AF (TGF-β1; Figure 5D). Together, these findings indicate that MMP12 is required for fibrotic NP remodeling and acquisition of myofibroblast-like cells in vivo. Moreover, removal of MMP12 under a degenerative state can promote a chondrogenic NP phenotype.

## Discussion

TGF-β1 is commonly regarded as a stimulator of chondrogenesis and a positive regulator of NP homeostasis (22). Previous study showed that local NPC could acquire a chondrogenic and later myofibroblastic phenotypes in puncture-induced IDD (14). Consistent with this finding, we found that human NPC could undergo myofibroblast differentiation in the presence of TGF-β. This implies that TGF-β function in IVD can be context dependent and that excessive TGF-β may promote NP fibrosis and, hence, IDD. Our data support MMP12 as a key mediator to control the differentiation under TGF-β signaling and, thus, regulate NP fibrosis.

Consistent with its role as a matrix-degrading enzyme, the loss of MMP12 may have encouraged the deposition of aggrecan and collagen II. Nevertheless, the loss of MMP12 also appears to accelerate disc height reduction in the early phase of injury-induced IDD. This may suggest that the mutants experienced a disrupted reparative response at early injury or degeneration stage. This finding is in line with the notion by Chen et al. that fibrosis might function to support disc mechanics at acute and subacute stages of injury-induced IDD (39). We therefore propose that the myofibroblast-like disc cells may be required to mediate a provisional reparative response in both NP and AF to compensate the acute loss of disc function. However, it is not clear if the persistence of myofibroblast-like cells in the WT injury-induced IDD resembles a dysregulated proliferative phase of defective wound healing. It is possible that at later stage of degeneration, the intrinsic microenvironment (e.g., chronic inflammation, hypoxia, and mechanical stress) may limit the repair, and the prolonged, excessive fibroblastic activity may eventually lead to NP remodeling and fibrosis. A time-course investigation of myofibroblast activity in various IDD models may further delineate their roles. In fact, MMP12 expression was also found in the endplate chondrocytes in the aging and degenerative sand rat discs (19). The function of endplate and growth plate might, therefore, also be disturbed in the *Mmp12^–/–^* mutants, leading to further destruction of disc microenvironment and mechanical homeostasis. It is interesting to note that our transcriptome RNA-Seq revealed DEGs (e.g., *ODAPH* and *IBSP*; ref. 33) enriched in ossification and cartilage development process in the *MMP12* knockdown cells. The contribution of MMP12 in the endplate chondrocytes warrants further investigation.

Our in vitro and in vivo data support a critical role of MMP12 in myofibroblast formation. However, the identity and origin of these myofibroblasts in IDD remain elusive. Au et al. previously reported that mouse notochordal cells can directly transit into chondrocyte-like and later fibroblastic cells in an injury-induced IDD model (14). We show here that SOX9^+^ and CNMD^+^ (2wpp) cells and later αSMA^+^ (12wpp) cells were induced in the NP in a similar IDD model. Lineaging tracing of NP-specific Mmp12-KO cells could help to define the role of MMP12 in the transition.

It should be noted that adult mouse discs contain distinct NPC composition from mature human discs. However, cumulative evidence supports an acquisition of similar cell heterogeneity between the mouse and human dNP, featuring an emergence of progenitors and other differentiated cells expressing fibroblast-like, epithelial, and monocyte/macrophage-like phenotype (4, 14, 20, 26–28). In this context, there is a likelihood that either human or mouse myofibroblast-like NPC are collectively derived from transdifferentiation of this resident pool of cells or other uncharacterized local or extrinsic populations. Our in vitro data demonstrate a reduction of epithelial cell, macrophage-like, and progenitor phenotypes in the TGF-β1–treated NPC. This implies that myofibroblastic cells might be derived from macrophages or disc progenitors and might be involved in epithelial-mesenchymal transition. The reduced *FAP* expression suggests that the degenerative cells contain fibroblast precursors (4), which may be involved in the myofibroblast-like differentiation. In line with this notion, TGF-β1 could reduce the expression of disc progenitor (*TEK2*) and mature NPC (*KRT19*) markers, and they increased *FAP* expression in non-degenerative cells (Figure 1B), indicating a potential of NPC in acquiring a myofibroblast-like phenotype via a fibroblast precursor stage. The presence of fibroblasts may further facilitate the generation and/or recruitment of myofibroblast. Transplantation of autologous fibroblasts into cynomolgus monkey IVD could induce NP fibrogenesis (39). Interestingly, Gan et al. identified a population of pericyte-like cells in healthy NP with expression of *ACTA2*, *TAGLN*, and *MCAM* (28). Given that mesoderm-derived pericytes (Ng2^+^, Pdgfrβ^+^) are considered as a major source of myofibroblasts (10), the possibility of pericyte-to-myofibroblast transition in IDD can be further investigated.

MMP12 could modulate lung and liver fibrosis via activating profibrogenic genes *EGR1* and *CYR61* (16, 17), oxidative stress induction (40), and limiting fibrillar collagen-degrading enzymes MMP2 and MMP9 (15). Alternatively, MMP12 could mediate EGFR and ERK1/2 activation, resulting in CXCL8 induction (41). Our transcriptome data also reveal the MMP12-dependent upregulation of *CYR61* and *CXCL8* in myofibroblast differentiation of human dNPC. The nuclear localization of MMP12 in NPC, especially after TGF-β1 treatment, implicates a function of MMP12 in transcriptional regulation, which has been reported in IκBα-mediated immunity (42). In fibroblastic NPC, MMP12 expression is downregulated by RhoA/MRTF-A inhibitor (43), implying the crosstalk of MMP12 and the RhoA/MRTF-A pathway in myofibroblastic NPC formation. In fact, MMP12 is traditionally regarded as an elastase secreted by macrophages. In addition to MMP12, the serine proteinases neutrophil elastase (NE) and other MMPs (e.g., MMP2/7/9) also mediate elastin degradation. MMP7 is reported to be a potent elastase with high activity in degenerative human disc (36). This may explain the unaffected elastin expression in the *Mmp12^–/–^* discs. Interestingly, MMP12-generated elastin fragments could serve as a self-antigen to drive the cigarette smoke–induced autoimmune processes (44). Whether this process plays a role in NP fibrosis, particularly in the early phase of IDD, awaits to be explored. Moreover, macrophages can undergo myofibroblast transition (CD68^+^αSMA^+^) via TGF-β signaling and support myofibroblast proliferation (45). In our study, MMP12 knockdown in the dNPCs could upregulate a macrophage-like phenotype, supporting the presence of macrophage-like cells and their myofibroblast transition in human IDD.

A limitation of this study is the use of non-tissue-specific *Mmp12* knockout mice. Despite no distinguishable phenotype during the skeleton maturation, Mmp12-null mice were reported to display immunosuppression (46), increased insulin sensitivity (47), and increased glucose metabolism (48), which may potentially influence the fibroinflammatory program of IDD. Furthermore, NPC are known to experience hypoxia, which links to NP homeostasis by balancing cellular autophagy and apoptosis, and a coordination of MMPs and TIMPs activity (49). Oxygen tension (≤5% O_2_) may regulate the chondrogenic activities of NPC and MSCs. Its effect on myofibroblast transition of NPC is yet to be examined.

In conclusion, the results of this study suggest MMP12-dependent myofibroblast formation as a key mechanism of NP fibrosis. We propose that the remodeling of NP is a coordinated dynamic process and can be independent of the degenerative events.

## Methods

### Sex as a biological variable.

Both female and male mice were included. In this study, sex was not considered as a biological variable.

### Culture of human BM-MSCs and NPC.

BM-MSCs were isolated using a standard extraction protocol by gradient centrifugation (300*g*) with multipotency confirmed by trilineage differentiation assay (Supplemental Figure 4). Human NP tissues were collected from symptomatic patients with lumbar IDD (age: mean **±** S.D = 50.7 ± 11.4; *n* = 6) undergoing spinal fusion procedures or adolescent patients with scoliosis (age: mean **±** S.D = 14.4 ± 1.34; *n* = 5) undergoing deformity correction surgery (Supplemental Table 2). Cells were isolated by sequential enzymatic digestion (20) and maintained in high-glucose DMEM (Thermo Fisher Scientific) containing 10% FBS supplemented with antibiotics. NPC at passage 4 were firstly cultured in alginate for redifferentiation and then released by calcium chelators (55 mM sodium citrate and 30 mM EDTA in 0.9% NaCl) to monolayer. Serum starvation was performed for 24 hours prior to TGF-β1 (Abcam, 10 ng/mL) treatment. BM-MSCs (less than passage 7) were similarly treated as a positive control (50).

### MMP12 siRNA transfection.

Human dNPC at 70%–80% confluence were transfected with MMP12 or scramble siRNA (synthesized by GenePharma, Suchow, China) for 24 hours using hyperfectamine (Qiagen) according to manufacturer’s instruction, followed by treatment with or without TGF-β1 (10 ng/mL) for 3 days. The sequences for MMP12-specific siRNA and the nontargeting siRNA were designed as previously described (51).

### Collagen gel contraction assay.

Myofibroblast contractility was investigated by modified collagen gel contraction assay (50). Briefly, 400 μL of rat collagen I gel solution (3 mg/mL in 1% acetic acid, Thermo Fisher Scientific) and 200 μL of cell suspension (100,000 cells/mL) were gently mixed, neutralized by NaOH, and maintained in 24-well plates. The gel constructs were cultured in complete DMEM as described above with or without TGF-β1 stimulation for 3 days, followed by incubation with MTT reagent for 1 hour. Gel constructs were imaged by digital camera (Z30, Nikon), and the magnitude of contraction was determined by differences of gel diameter.

### Western blotting.

Unless specified, antibodies were raised in rabbit and purchased from Abcam. Protein lysates were extracted in RIPA buffer and quantified by Bradford assay (Bio-Rad). Equivalent amounts of protein were resolved by 10% SDS-PAGE and transferred to PVDF membranes (Bio-Rad). Membrane was blocked in 7.5% BSA in TBST buffer for 1 hour at room temperature and probed with the primary antibodies (Supplemental Table 3) overnight at 4°C. Antibody binding was detected by secondary peroxidase-conjugated goat anti–rabbit IgG (GB23303, Servicebio) and visualized by chemiluminescence.

### Quantitative PCR.

Total RNA was isolated from the cells using TRIzol plus RNA purification kit (Invitrogen). Reverse transcription was performed with a high-capacity cDNA reverse transcription kit (Thermo Fisher Scientific) according to the manufacturer’s protocol. The cDNA for the myofibroblast markers genes was amplified using the primers described in Supplemental Table 4. *GAPDH* was used as the endogenous control, and relative expression levels were determined by the comparative ΔΔCt method.

### Bulk RNA-Seq and bioinformatic analysis.

The total RNA, isolated as described above, were treated with DNase I followed by PCR amplification and library construction. Primary sequencing was performed using Illumina NovaSeq 6000 sequencer (Illumina Inc.) with clean reads aligned to the hg19 human reference genome. All samples were amalgamated to reconstruct a comprehensive transcriptome. Differentially expressed genes (DEGs) between nontreated and TGF-β1–treated NPC — or between TGF-β1–treated NPC with MMP12 siRNA transfection and scramble siRNA — were identified using the “DESeq2” package in R package (version 4.2.0) with a FDR-adjusted *P* < 0.05 and log_2_ fold change > 0.585 or 1. Gene Ontology (GO) and the Kyoto Encyclopedia of Genes and Genomes (KEGG) analyses were performed using enrichGO and enrichKEGG functions in R package clusterProfiler.

### Induction of mouse tail IDD by annulus puncture.

Skeletally matured (4–6 months old) Mmp12-null (*Mmp12^–/–^*) mice (female/male [F/M], *n* = 10, B6.129X-Mmp12^tm1Sds^, The Jackson Laboratory) in C57BL/6J background received annulus puncture in the tail discs at CC6/7 level. Age-matched C57BL/6J mice were used as WT control (*n* = 10). Mouse disc levels were identified under X-ray (model 43855a; Faxitron). Annulus puncture was implemented via perpendicular penetration of a 27 G needle from dorsal side at 5 mm depth into the middle of disc, rotated 180°, and held for 30 seconds. After removal of the needle, the skin was sutured, and the mice underwent standard postoperative care.

### Disc height analysis.

Anteroposterior spine radiographs were taken for the mice at 1, 2, 4, 8, and 12 weeks after surgery. The height of each disc was calculated by 2 independent observers and represented as disc height index (DHI) as previously described (14).

### Histological analysis and immunostaining.

The segments of punctured (CC6/7) and unoperated (CC7/8) discs with adjacent vertebral bodies were harvested at 2 and 12 weeks after surgery, fixed, and decalcified in 22% formic acid (in 10% sodium citrate). After routine dehydration procedures, specimens were embedded in paraffin and sectioned for multichromatic FAST staining (14) and Picrosirius red (39). After Picrosirius red staining, collagen fiber was visualized under polarized light microscopy and fiber metrics analysis was performed by CT-FIRE (http://loci.wisc.edu/software/ctfire). Degenerative changes were quantified by histological scoring (37). For immunostaining, sections were incubated for 4–6 hours at 60°C with citrate buffer (10 mM citrate, 0.05% Tween [pH 6.0]) for antigen retrieval. Signals for TGF-β1 (ab215715, Abcam), αSMA (ab5694, Abcam), MMP12 (PA5-13181, Invitrogen), SOX9 (ab185230, Abcam), CNMD (PA5-76974, Invitrogen), aggrecan (ab315486, Abcam), elastin (PA5-99418, Invitrogen), and collagen I (COL1 PA1-26204, Invitrogen), II (COL2 PA5-99159, Invitrogen), and III (COL3 ab7778, Abcam) were detected by polyclonal rabbit antibody (1:200; Supplemental Table 3) and observed by immunostaining using AP-conjugated secondary antibody from ImmPRESS duet double staining kit (Vector Labs) based on the manufacturer’s instruction or immunofluorescence using Alexa Fluor 594–conjugated secondary antibody (GB28301, Servicebio). DAPI was used for nuclei counterstaining. Virtual whole disc stain acquisition was accomplished in Photoshop (Adobe) at high resolution (200×). For αSMA, SOX9, and CNMD staining, positive cells were determined by counting of 5 independent samples. Image assessments were conducted by 2 independent observers who were blinded with regard to treatment group. Cultured NPC were fixed in 10% ice-cold methanol for 5 minutes, permeabilized with 0.1% Triton-x-100 for 10 minutes at room temperature, and incubated with 7.5% BSA for 1 hour at room temperature. MMP12 was detected by binding with primary antibody (PA5-13181, Invitrogen) overnight at 4°C followed with Alexa Fluor 594–conjugated secondary antibody (GB28301, Servicebio) for 1 hour at room temperature. Sections were mounted with DAPI and checked under fluorescence microscope.

### Statistics.

Data were presented as mean ± SD. All the experiments were repeated for at least 3 times. For gene expression in *MMP12* siRNA transfection, Brown-Forsythe and 1-way Welch ANOVA with Dunnett’s post hoc test was performed. Two-way ANOVA with Bonferroni’s multiple-comparison test was conducted for protein expression quantification in *MMP12* siRNA transfection, DHI and immunofluorescence cellularity analysis data. For normalized gene/protein expression data, 1-sample, 2-tailed *t* test was employed instead. Two-tailed unpaired *t* test was performed in gel contraction assay. *P* values less than 0.05 were considered significant.

### Study approval.

In this study, human BM-MSCs and NPC were harvested under the approval of the University of Hong Kong IRB with patient consent. Animal experiment protocols were approved by the Committee on the Use of Live Animals in Teaching and Research of the University of Hong Kong (CULATR 5443).

### Data availability.

All relevant data supporting the findings in this study are available within the article and its supplemental files. Values for all data points are reported in the Supporting Data Values file. RNA-Seq raw fastq files are deposited under the Gene Expression Omnibus (GEO) accession no. GSE286069. Experimental materials can be accessed upon request to the corresponding authors via a Data Transfer Agreement.

## Author contributions

YS, XZ, and VYL contributed to the study design and obtained fundings for the study. YS conducted the bioinformatic analysis. YS and WKT performed the in vitro and in vivo experiments and data analysis. PC, PZ, Y Huang, and ZZ collected samples and clinical data. YS, MZ, QL, MY, Y Hsu, Y Huang, and ML conducted the human NPC isolation and culture. YS and VYL wrote the manuscript. WKT, ZZ, XZ, and VYL reviewed the manuscript. VYL conceptualized and supervised the whole project, provided support, and coordinated the collaboration. All authors have read and approved the manuscript.

## Supplementary Material

Supplemental data

Unedited blot and gel images

Supporting data values

## Version 1 03/04/2025

In-Press Preview

## Version 2 04/08/2025

Electronic publication
