# Supplementary material for: MMP12-dependent myofibroblast formation contributes to nucleus pulposus fibrosis
Source: JCI Insight. 2025 Mar 4;10(7):e180809. doi: 10.1172/jci.insight.180809 (PMC11981621; doi:10.1172/jci.insight.180809)
Supplement: Supplemental data [file jciinsight-10-180809-s073.pdf]

Supplementary Figures

Supplementary Figure S1. Immunostaining of TGF-β1, MMP12, elastin, αSMA, Sox9, collagen I (COL1), II (COL2) and III (COL3), and aggrecan in un-operated discs of wild type (*w.t*) and *Mmp12* knockout (*Mmp12*$^{-/-}$) mice.

Representative images are shown. Scale bar: 200um.

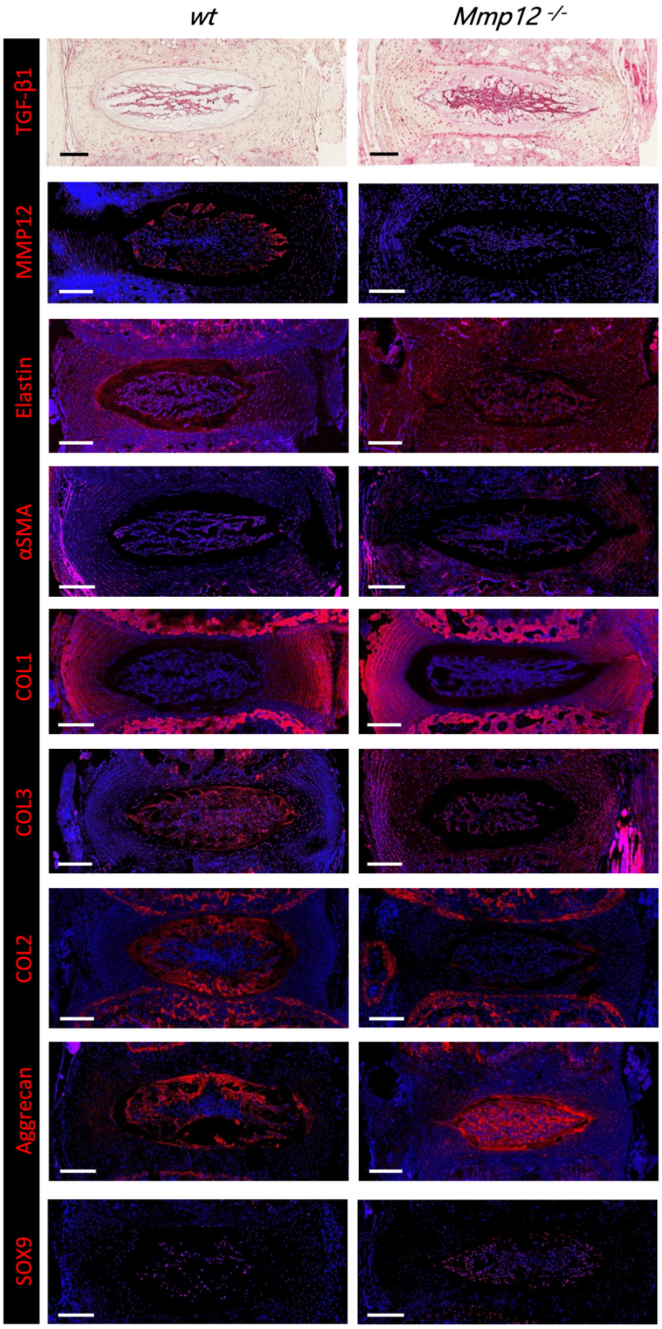

Supplementary Figure S2. Histological scoring details.

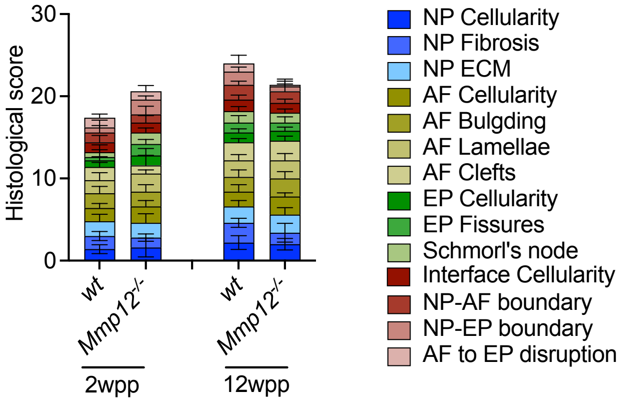

Supplementary Figure S3. Quantification of collagen fiber metrics in annulus fibrosus. (A) Abundance; (B) Density; (C) Length of collagen fibers in AF from punctured disc were calculated by CT-FIRE based on Sirius red stained sections. IOD, integral optical density.

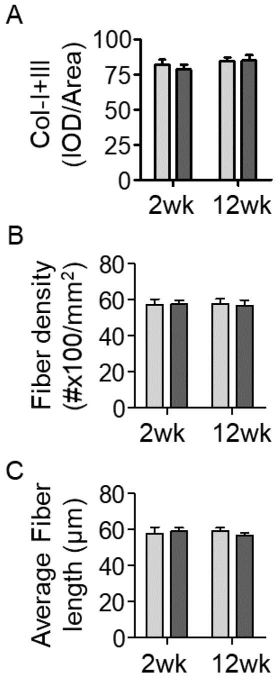

Supplementary Figure S4. Tri-lineage differentiation of MSC. Bone marrow derived mesenchymal stromal cells (MSC) were cultured in basal growth medium (G.M) or medium for chondrogenic (C.M), adipogenic (A.M) or osteogenic (O.M) differentiation (n=3). Chondrogenic differentiation was assessed by protein (A) and gene (B) expression of Sox9, aggrecan and collagen II. (C) Adipogenic differentiation was assessed by oil-red staining. (D) Osteogenic differentiation was assessed by alkaline-phosphatase (ALP) staining. * $p<0.05$; ** $p<0.01$; *** $p<0.001$ determined by two-way ANOVA with Bonferroni post-hoc test for gene expression or unpaired t test for oil-red and ALP quantification.

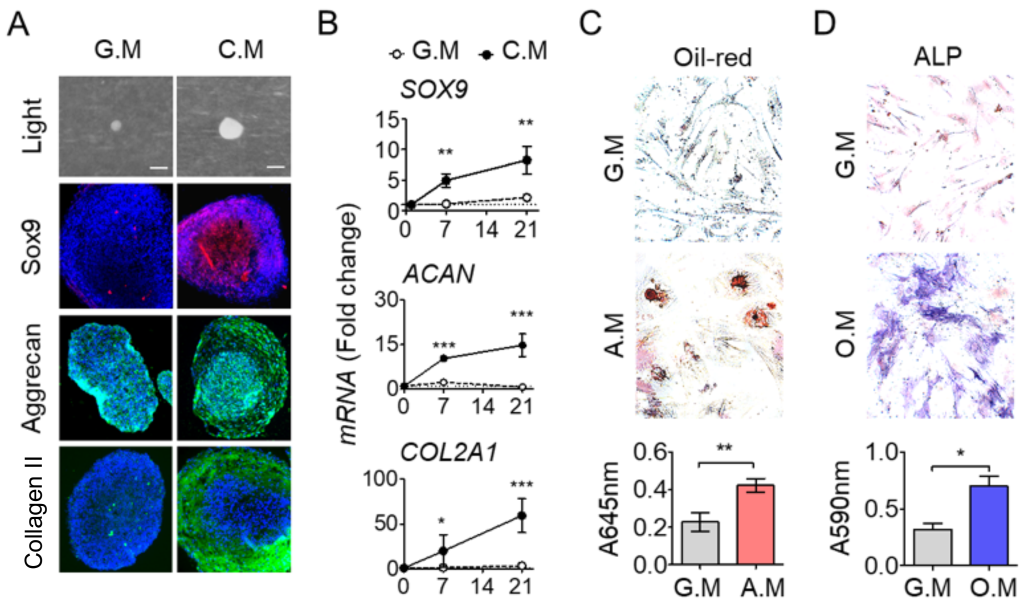

Supplementary Table 1. Summary of DEGs fold changes of biomarkers for NPC subtypes. Biomarkers were defined based on previous single-cell RNA transcriptome data. DEGs fold changes were extracted from dNPC transcriptome.

**NPC subtype**	**Signature genes**	**TGF-β1 treatment**		**TGF-β1 + siMMP12 transfection**	
		Log10 Fold changes	*p* value	Log10 Fold changes	*p* value
**ProgNP**	*SERPINF1*	-0.35353	0.02179	-0.06939	0.60959
	FBLN1	-0.76456	0.00000	0.25278	0.00371
	SERPING1	-0.81262	0.00000	-0.00544	0.94846
	PLA2G2A	-1.15865	0.04598	-0.38472	0.51976
	GPX3	-0.81740	0.00000	0.13232	0.03339
	IGFBP6	-0.68152	0.00000	-0.02606	0.78845
	LTBP4	0.06662	0.69464	0.20667	0.01639
	APOD	-0.79058	0.00000	0.11812	0.44944
	TNXB	-1.66075	0.00000	0.55348	0.52363
	PDGFRA	-1.01243	0.00000	0.23163	0.00499
**ChonNP**	*COL2A1*	0.05356	0.84048	0.03651	0.79089
	SNORC	-0.29062	0.22196	0.17504	0.49382
	cnmd	-0.38077	NA	-0.28583	0.73431
	caps	-0.42344	0.01422	0.18426	0.29290
	COL9A3	-0.78564	0.00245	-0.15518	0.37372
	SERPINA5	-0.20289	0.39099	0.55912	0.00000
	ACAN	-0.87870	0.00000	0.24575	0.01275
	COL9A3	-0.78564	0.00245	-0.15518	0.37372
	SERPINA3	-0.24034	0.02648	0.10494	0.12232
	CHRDL2	-0.75105	0.00346	0.31223	0.15477
**FibroNP**	*COL1A1*	0.47739	0.00005	-0.20794	0.00500
	COL3A1	0.68955	0.00003	-0.59605	0.05452
	fn1	0.75311	0.00000	-0.26726	0.00023
	POSTN	0.91195	0.00000	-0.17097	0.05013
	SPARC	0.56591	0.00000	-0.21334	0.00182
	MMP2	0.63070	0.00000	-0.08730	0.57110
	TNC	-0.19623	0.13486	-0.37129	0.00000
	SERPINH1	0.40708	0.00000	-0.19458	0.00111
	COL1A2	0.18973	0.10765	-0.16625	0.03501
	PRSS23	0.17911	0.02630	-0.11760	0.00741
**CyclingNP**	*STMN1*	-0.03450	0.90479	-0.55010	0.01575
	PTTG1	0.12637	0.70262	-0.29985	0.09727
	TOP2A	0.16719	0.73264	-0.97406	0.03677
	CENPF	0.18771	0.69962	-0.45732	0.27032
	NUSAP1	0.14769	0.77907	-0.48655	0.17594
	LGALS1	0.32682	0.00001	-0.14202	0.04842
	TUBB	0.41352	0.18634	-0.05530	0.84679

	BIRC5	0.13316	0.80275	-0.51180	0.23991
	TYMS	-0.07246	0.76884	0.09672	0.49409
	SMC4	-0.13361	0.19491	-0.24311	0.00034
**RegNP**	*MMP3*	-0.18043	0.45491	0.15506	0.22159
	CHI3L1	-1.29153	0.00000	0.03514	0.86673
	GPX3	-0.81740	0.00000	0.13232	0.03339
	BMP2	-0.33695	0.28731	-0.01535	0.91952
	SOD2	-1.03172	0.00000	-0.15638	0.03082
	C11orf96	0.18211	0.05736	-0.30105	0.00277
	SLC7A2	-0.88895	0.00000	0.18498	0.12220
	SERPINE2	0.32991	0.08224	0.22788	0.00739
	NFKBIZ	0.45020	0.00000	-0.03879	0.58694
	IER3	0.95232	0.00006	-0.15880	0.34601
**Macrophage**	*CD74*	-1.48558	0.00011	-0.31001	0.15669
	CD68	-0.53701	0.09209	0.27243	0.00164
	CCL3L1	-0.71028	NA	0.49116	0.65090
	HLA-DRB1	-0.14499	0.83996	0.33236	0.42207
	IL1B	NA	NA	1.05518	0.11622
	CXCL3	-1.19083	0.03538	-0.34991	0.55653
	LYZ	-0.28907	NA	NA	NA
	CXCL8	-0.85258	0.00289	0.19976	0.49503
	TYROBP	-0.35837	NA	NA	NA
	HLA-DPA1	-0.84383	0.23701	1.14710	0.33285
**Endothelial cell**	*SPARCL1*	-0.84404	0.06955	-0.50420	0.26285
	IFI27	-0.63983	0.40687	1.53170	0.00732
	PECAM1	-0.35529	0.48922	-0.12910	0.73075
	PLVAP	-0.14245	0.84491	0.37112	0.36893
	SYNE2	-0.64702	0.00000	0.20163	0.03573
	CALCRL	0.10744	0.64821	0.42068	0.00639
	IGFBP4	-0.42947	0.00000	0.24125	0.00057
	VWF	-0.85639	0.00322	0.71970	0.02028
	ITGA6	-0.10931	0.48464	0.03303	0.79554
	CD34	-0.50161	0.16563	0.28226	0.44814

Supplementary Table 2. Demographic data. A total of 11 subjects were recruited with an age between 13 – 71 (average = 34.2). Grade of disc degeneration was determined by MRI according to Pfirrmann criteria. F, female; M, male; IS, idiopathic scoliosis; IDD, intervertebral disc degeneration; ND, not determined.

Sample	Sex	Donor's Age (Years)	Level	Disease	Grade
1	F	13	L2-3	IS	ND
2	F	13	L2-3	IS	ND
3	M	15	L2-3	IS	ND
4	F	15	L2-3	IS	ND
5	F	16	L1-2	IS	ND
6	M	48	L4-L5	IDD	IV
7	M	40	L3-L4	IDD	III
8	F	42	L4-L5	IDD	IV
9	F	47	L3-L4	IDD	V
10	F	56	L4-L5	IDD	III
11	M	71	L4-L5	IDD	IV

Supplementary Table 3. Antibody information.

Description	Sources	Product Cat #
MMP12 Polyclonal Antibody	Invitrogen	PA5-13181
Elastin Polyclonal Antibody	Invitrogen	PA5-99418
Rabbit polyclonal to SOX9	Abcam	ab185230
LECT1 Polyclonal Antibody	Invitrogen	PA5-76974
Rabbit polyclonal to alpha smooth muscle Actin	Abcam	ab5694
Rabbit polyclonal to Fibroblast activation protein, alpha	Abcam	ab53066
Rabbit polyclonal to CYR61	Abcam	ab286129
Rabbit recombinant multiclonal [RM2036] to Aggrecan	Abcam	ab315486
Collagen I Polyclonal Antibody	Invitrogen	PA1-26204
Rabbit polyclonal to Collagen III	Abcam	ab7778
Collagen II Polyclonal Antibody	Invitrogen	PA5-99159
Rabbit polyclonal to GAPDH	Abcam	ab9485

Supplementary Table 4. Primer sequences for RT-PCR.

Gene ID		Sequence, 5’–3’
ACTA2	Sense	CTATGAGGGCTATGCCTTGCC
	Antisense	GCTCAGCAGTAGTAACGAAGGA
COL1A1	Sense	ATCAACCGGAGGAATTTCCGT
	Antisense	CACCAGGACGACCAGGTTTTC
COL3A1	Sense	TTGAAGGAGGATGTTCCCATCT
	Antisense	ACAGACACATATTTGGCATGGTT
MMP12	Sense	GGAATCCTAGCCCATGCTTTT
	Antisense	CATTACGGCCTTTGGATCACT
TEK	Sense	TCCGCTGGAAGTTACTCAAGA
	Antisense	GAACTCGCCCTTCACAGAAATAA
KRT19	Sense	ACCAAGTTTGAGACGGAACAG
	Antisense	CCCTCAGCGTACTGATTTCCT
FAP	Sense	CAAAGGCTGGAGCTAAGAATCC
	Antisense	ACTGCAAACATACTCGTTCATCA
ITGB6	Sense	TCCATCTGGAGTTGGCGAAAG
	Antisense	TCTGTCTGCCTACACTGAGAG
CYR61	Sense	CTCGCCTTAGTCGTCACCC
	Antisense	CGCCGAAGTTGCATTCCAG
GAPDH	Sense	GGAGCGAGATCCCTCCAAAAT
	Antisense	GGCTGTTGTCATACTTCTCATGG
