# Supplementary figures and images for: MMP12-dependent myofibroblast formation contributes to nucleus pulposus fibrosis
Source: JCI Insight. 2025 Mar 4;10(7):e180809. doi: 10.1172/jci.insight.180809 (PMC11981621; doi:10.1172/jci.insight.180809)

Un-cut gel images for WB from  
#Figure 1B

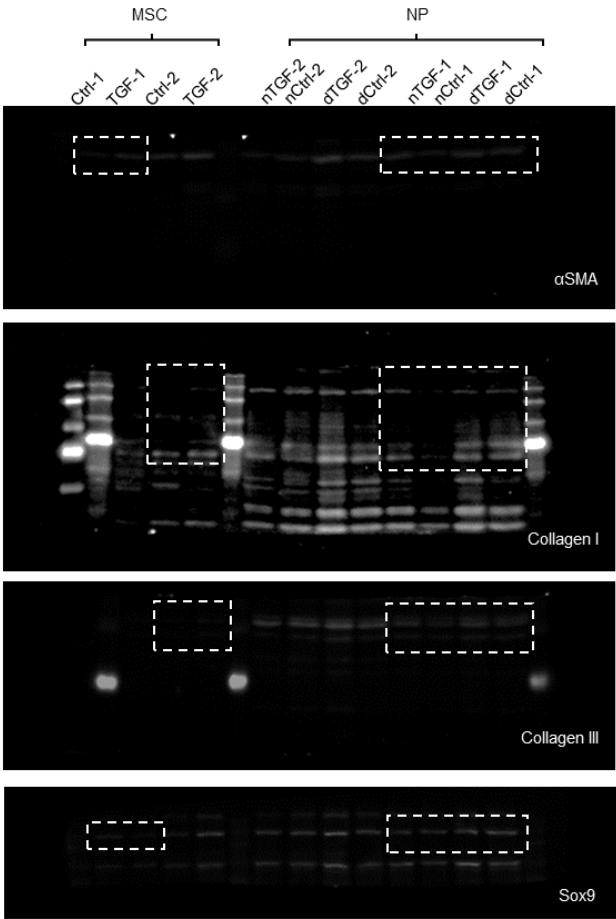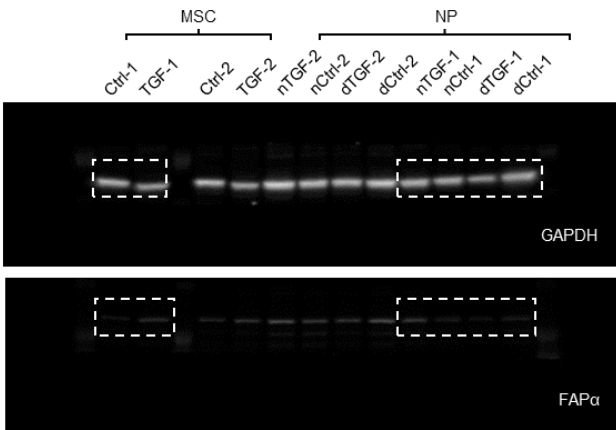

#Figure 3A

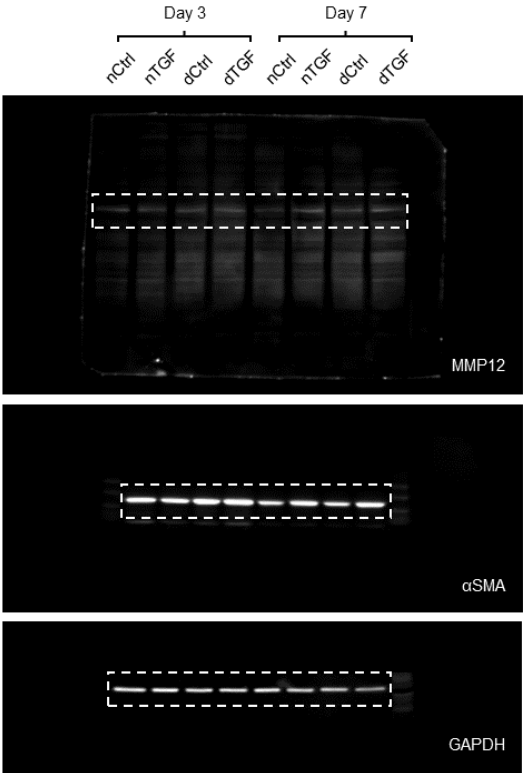

#Figure 3D

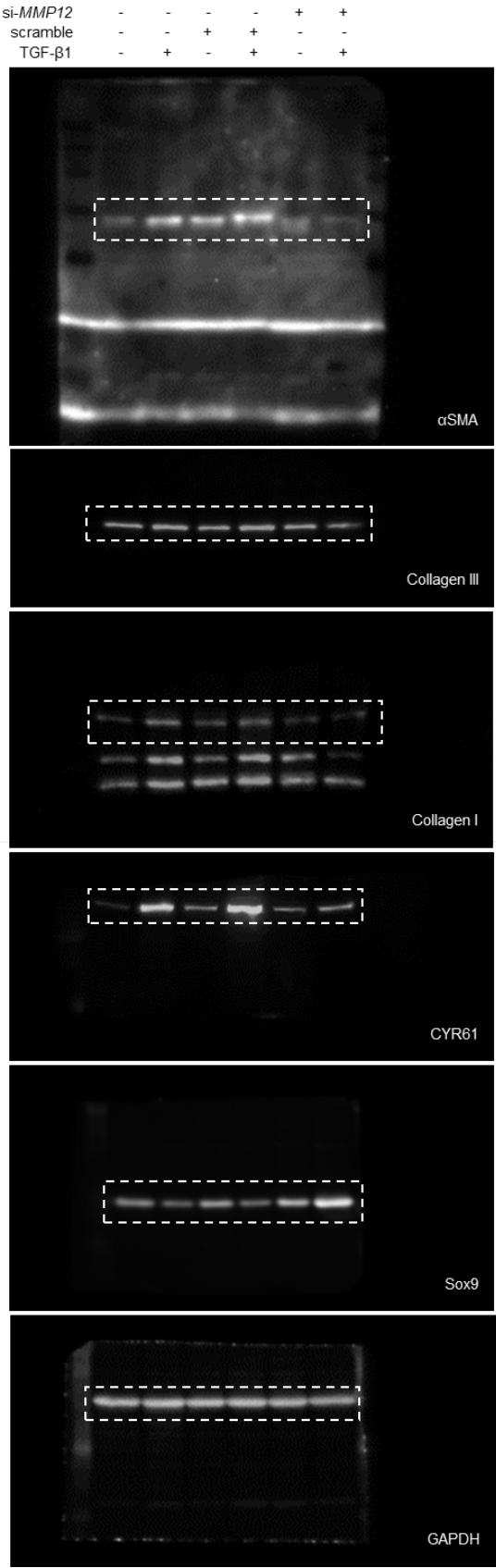

#Figure 3D

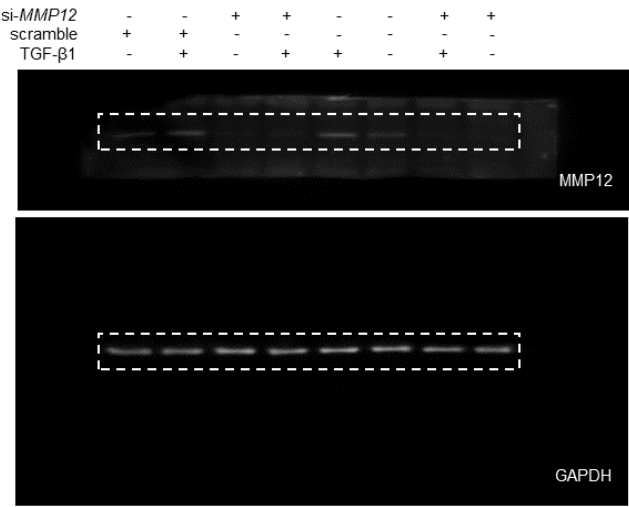

Supplement: Unedited blot and gel images [file jciinsight-10-180809-s074.pdf]
